# A comprehensive review of pancreatic cancer and its therapeutic challenges

**DOI:** 10.18632/aging.204310

**Published:** 2022-09-28

**Authors:** Shan Jiang, Johan Bourghardt Fagman, Yunyun Ma, Jian Liu, Caroline Vihav, Cecilia Engstrom, Beidong Liu, Changyan Chen

**Affiliations:** 1 Institute of Clinical Sciences, University of Gothenburg, Gothenburg, Sweden; 2 Institute of Clinical Sciences, Sahlgrenska Academy, University of Gothenburg and Sahlgrenska University Hospital, Gothenburg, Sweden; 3 The First Affiliated Hospital of Nanchang University, Nanchang, PR China; 4 Department of Chemistry and Molecular Biology, University of Gothenburg, Gothenburg, Sweden

**Keywords:** pancreatic cancer, etiology, progression, genetic mutations, therapeutics

## Abstract

Pancreatic cancer is a devastating and lethal human malignancy with no curable chemo-treatments available thus far. More than 90% of pancreatic tumors are formed from ductal epithelium as pancreatic ductal adenocarcinoma (PDAC), which often accompany with the expression of mutant *K-ras*. The incidences of pancreatic cancer are expected to increase rapidly worldwide in the near future, due to environmental pollution, obesity epidemics and etc. The dismal prognosis of this malignancy is contributed to its susceptibility to tumor micro-metastasis from inception and the lack of methods to detect precursor lesions at very early stages of the onset until clinical symptoms occur. In recent years, basic and clinical studies have been making promising progresses for discovering markers to determine the subtypes or stages of this malignancy, which allow effectively implementing personalized therapeutic interventions. The purpose of this review is to discuss the existing knowledge of the molecular mechanisms of pancreatic cancer and the current state of treatment options with the emphasis on targeting therapeutic approaches. The specific focuses are on the molecular mechanisms of the disease, identifications of drug resistance, establishment of immune escaping mechanisms as well as potential of targeting identified pathways in combinations with existing chemo-drugs.

## INTRODUCTION

The number of incidences of pancreatic cancer is very similar to that of its deaths, and therefore this malignancy is considered aggressive and deadly. Although some improvements and progresses have been made for the diagnosis or treatments, the projected 5–6 years of survival from pancreatic cancer has not significantly changed, and there is an expectation of rising incidences in the near future [1, 2]. There are several features of pancreatic cancer responsible for its dismal prognosis. For example, the malignancy is often diagnosed in its late stages when clinic symptoms appear. The disease frequently undergoes micro-metastasis from inception, which causes a poor prognosis even for those cases noticed at early stages. Furthermore, low densities of vasculatures and formation of fibrotic barrier in pancreatic cancer tissue areas impair the penetration of chemo-drugs and help build up drug resistance. Clearly, better understandings of molecular underpinnings of pancreatic cancer are important for developing novel diagnostic methods and efficient therapeutics.

Environmental factors and high-fat diet, smoke and alcohol appear to play significant roles in promoting pancreatic cancer risk and onset. The majority of the incidences of pancreatic cancer occur sporadically and the disease is often accompanied with genetic abnormalities in somatic cells. Studies have indicated that unlike familial cancer syndromes for gastro –intestine/colon or breast, pancreatic cancer has very low penetrance with less than 10% of the cases to be linked to a familial setting [3, 4]. Among many genetic lesions detected in pancreatic cancer, active mutant *K-ras* is one that occurs in early stages of pancreatic tumorigenesis, followed by inactivation of several tumor suppressors, including *p53, p16^INK4a^*, and others (such as *SMAD4/DPC4, MLH1*, *LKB1, PRSS1*, and *BRCA2*) [3, 4].

Enormous basic research efforts have been focusing on exploring immunosuppression in pancreatic cancer, searching for targets to disrupt the barrier of tumor microenvironment and developing personalized animal models of the disease. Progress has been made in therapeutics by identifying mutations of pancreatic cancer signature genes for developing more precisely targeted treatments [5, 6]. More recently, immunotherapies are one of the novel strategies for pancreatic cancer, some of which are included in clinical trials [7]. It is anticipated that more effective targeted and personalized strategies will be generated via bench investigations and refined animal models. Here, we summarize the existing information and provide an overview of the underlying mechanisms of pancreatic cancer onset and progression as well as the current clinical development.

## Pancreatic cancer etiology and progression

With aging, the risk of the onset of pancreatic cancer, particularly PDAC, is increasing. Among the patients, less than 10% appear having an inherited predisposition, which is in a familial setting with a low penetrance [8, 9]. Besides the possible link to genetic abnormality, factors that contribute to the pathology of pancreatic tumors have generally been shown to be pancreatitis, bad diet habits, tobacco smoking (possible e-cigarette smoking), and excessive alcohol consumption [10–13].

### Genetic alterations during pancreatic tumorigenesis

Biological and biochemical analyses have demonstrated high genomic complexity in pancreatic cancer, in which mutations are frequently detected in somatic cells, and the tumors present highly heterogeneous with various genetic changes [14, 15]. These studies reveal high frequencies of genetic alterations in the tumors, which are noticed as changes of expressions of tumor suppressor genes and oncogenes, such as *K-ras*, *T53*, *p16^INK4A^/ARF*, *MLH1*, *LKB1, PRSS1*, and *BRCA2* [16–18]. Among these genetic changes, we here focus on describing the oncogenic *K-ras* and, tumor suppressors’ *p53* and *p16^INK^* (*CDKN2, MTS1*).

Mutant *K-ras* is detected in more than 90% of pancreatic cancer patients and considered as one of the possibly early elements in pancreatic cancer pathogenesis [16, 19–21]. *K-ras* is located on chromosome 12 and its encoded protein belongs to the Ras family of GTP-associating proteins. Ras family consists of three major forms, K-, H. and N-Ras and the active form of Ras transmits signals to promote various cellular activities, including cell growth, differentiation, survival and apoptosis under certain circumstances. In most of pancreatic cancer, the single mutation is detected at the position G12 of the amino acid sequence of K-Ras, which is substituted glycine to aspartic acid or valine [22, 23]. Less frequent missense mutations of *K-ras* are at the codons of 13, 59, 61 or 63 [24–26]. Balletic mutations of this oncogene are also revealed by deep sequencing of exomes [27]. Studies reveal the peri-ductal lymphocyte infiltration and gastric mucous neck cell hyperplasia in the pancreases of the genetically modified mutant *K-ras* mice [28, 29]. Furthermore, the specific mice are generated by crossing the knock-in mice in which a *Cre*-activated *Kras^G12D^* is knocked into the endogenous *K-ras* locus with mice expressing *Cre* recombinase that is expressed by a *Pdx1* (pancreatic islet specific) or *Ptf1-p48* (pancreatic acinar specific) [30–32]. These transgenic mice express *Cre* recombinase under the control of the mouse *Pdx1* (pancreatic and duodenal homeobox 1) promoter. Mosaic Cre recombinase activity is detected in the pancreatic epithelium, antral stomach and duodenum in neonates and in pancreatic beta islet cells in adults. Specifically, early lesions in the mice are detected, in which the Notch pathways is noticeably activated with over-expressions of cyclooxygenase-2 (COX-2) or MMP-7, sometimes accompanied with metastasis. Thereafter, approximately one year after the development of PanIN, some of these genetic engineered pancreatic cancer mice develop pancreatic cancer (mainly PDAC), in which the origin of the tumors appears from acinar cells or acinar precursors. The spontaneous knockout or mutations of *p53* or *p16* permits a full penetrance of the cancer in the animals. Thus, these studies suggest the close connection among mutant *K-ras*, PanIN and pancreatic tumors.

Mutations in *K-ras* prohibit its encoded onco-protein to associate with and further be inhibited by GTPase-activating proteins (GAPs), leading to mutant Ras staying in a persistently or constitutively active status. As the result, multiple downstream effector pathways of Ras, such as Raf/ERK1/2/MEK or PI3K/Akt, are activated for promoting uncontrollable cell growth, desensitizing cell death, remodeling cellular metabolism, escaping from immune surveillance and increasing cell invasion. Studies showed that pancreatic tumor cells can be shed and further circulate in the blood stream. Therefore, mutant *K-ras* can be detected in circulating tumor cells, which has been used to facilitate clinical diagnostic imaging analyses [33].

*p53* is located on chromosome 17 and its encoded protein serves as a tumor suppressor for protecting the integrity of the genome of cells. This tumor suppressor is often inactivated or mutated in about 70% of pancreatic cancer and most of mutations are the missense mutations, especially at the locus of R248, R273 or R175 [34]. Using animal models, studies demonstrate that the loss of heterozygosity (LOH) of p53 is an important factor for driving pancreatic cancer progression [35, 36]. Specifically, loss of p53 appears to cooperate with oncogenic *K-ras*-induced pancreatic cancer initiation and progression, by perturbing cell cycle progression, impairing DNA damage repair, augmenting survival activities and hindering apoptosis in cells [35, 36]. *p53* mutations are more commonly detected in mutant *K-ras* pancreatic tumors than that expressing wild-type *p53*, suggesting that *K-ras* mutations developed in early stages of pancreatic tumorigenesis, creates a genetic background favoring *p53* mutations. The cooperation of these two oncogenes or proteins promotes uncontrollable cell growth, cell cycle progression, improper damage repair and establishment of genetic instability, which promotes pancreatic tumorigenesis [35, 36]. *p16^INK^* (*CDKN2, MTS1*) is another tumor suppressor and its inactive form is found in a large number of pancreatic cancer patients [16, 37]. This suppressor gene is anchored on chromosome 9 and the encoded protein regulates the cell cycle by preventing cells from improperly entering the S phase through inhibiting cyclin-dependent kinase (CDK) 4/6. Due to the inactivation of *p16^INK^* by the promoter methylation, missense mutation and deletion, its related cell cycle checkpoint is perturbed, which allows pancreatic cancer cells to improperly progress from G_1_ to S phase without repairing potential damages. As the result, risks of genetic instability in cells are increased. This is further demonstrated by the animal study that the knockout of *p16^INK^* causes the deregulation of the cell cycle transition and rapid advance of pancreatic tumorigenesis a *K-ras* transgenic mouse strain [38]. Overall, mutations of *p16^INK^* and *p53* are frequently observed in pancreatic tumors and the linear relationship of these two tumor suppressors in pancreatic tumorigenesis remains unclear.

Epigenetic abnormalities that alter DNA methylation, histone modification or microRNA expression are other factors to change gene functions in driving and promoting pancreatic tumorigenesis [32]. In some pancreatic cancers, tumor suppressor or DNA repair genes (such as *CDKN2A, CDH1 and MLH1*) are found to be silenced by methylation [16]. The over-expressions of microRNAs in pancreatic cancer have also been revealed, which seemed to participate in pancreatic neoplastic development [39–44].

### Pathological precursors of pancreatic cancer and its subtypes

During the development of cancer in the pancreases, the conventional model of the progression of pancreatic cancer suggests that early genetic changes initiate tumor-prone activities in a cell or few cells that then undergo clonal expansion to achieve a full transformation. Another scenario is that pancreatic cancer cells disseminate early and then undergo transformation independently [38]. It is also being suggested that pancreatic cancer is originated from acinar cells that undergo the process of the acinar-to-ductal metaplasia (ADM), during which *K-ras* mutations are acquired [39–41]. Overall, genetic-initiated alterations are considered as the bases for the classification of different subtypes of pancreatic tumors. In recent years, using advanced biological techniques, such as genomic, transcriptomic and proteomic assays that enable to identify different characteristic clusters of tumor cells with different gene expressions and mutations, studies demonstrated that pancreatic tumors can be divided into different subtypes [39–41]. In particular, global gene expression analysis reveals three subtypes of PC as: classical, quasi-mesenchymal, and exocrine-like subtypes [39]. It also pointed out that the prognosis and therapeutic responses of these subtypes are different. The classical subtype of pancreatic cancer expresses high levels of adhesion-associated and epithelial genes. The quasi-mesenchymal subtype shows an augmented expression of mesenchyme-related genes. The exocrine-like subtype contains the tumor cells that express high levels of digestive enzyme genes. Three metabolic subtypes were also identified in pancreatic cancer patient samples by metabolomics analysis, as slow proliferating, glycolytic and lipogenic subtypes [42]. The classifications of the subtypes of the cancer by these methods correlate well with each other. In addition, the analyses of the genomic sequencing plus copy number variation measurement demonstrated the mutation landscapes of the cancer and four subtypes are accordingly classified as stable, locally rearranged, scattered and unstable [43]. The tumor-specific and stroma-specific subtypes of pancreatic cancer were also classified by RNA sequencing and computational analysis [44]. It is noticeable that various immunological features of the tumors are recently included for determining the tumor subtypes [41]. Taken together, the uses of current available modern techniques permit better understandings of pancreatic cancer subtypes at molecular levels and more accurate predictions for outcomes of treatments.

Although advanced technology helps us obtain the molecular insight into pancreatic cancer, it still remains enigma about the association of PanINs to the onset of pancreatic cancer, as inflammatory lesions are frequently detected in pancreatic cancer patients in early stages of the malignancy, but not in all PanIN patients. It is known after pancreatic inflammation or injury, acinar cells in pancreatic ducts start to lose their properties gradually and form lesions with changes around the ductal, which can be categorized pathologically in four grades (PanIN 1A, 1B, 2 and 3) [16, 38, 45]. The lesions of PanIN 1 are recognized by consisting of columnar epithelial cells with basally aligned nuclei [46]. The lesions are flat as PanIN 1A or papillary as PanIN 1B. PanIN 2 lesions have more changes in the nuclei manifested as loss of nuclear polarity, nuclear pleomorphism, hyperchromasia, or pseudo-stratification. In PanIN 3 lesions, a large degree of dysplasia exists, which alter architectures, such as formation of papillae or clusters of cells from the epithelium invading into the lumen of the duct, accompanied with various nuclear changes. Therefore, current research approaches for obtaining better defining subtypes, mutations of the cancer, together with external elements, certainly provide better distinguish of benign PanINs from those inflammation-associated neoplasia or tumorigenesis.

**Figure 1:**
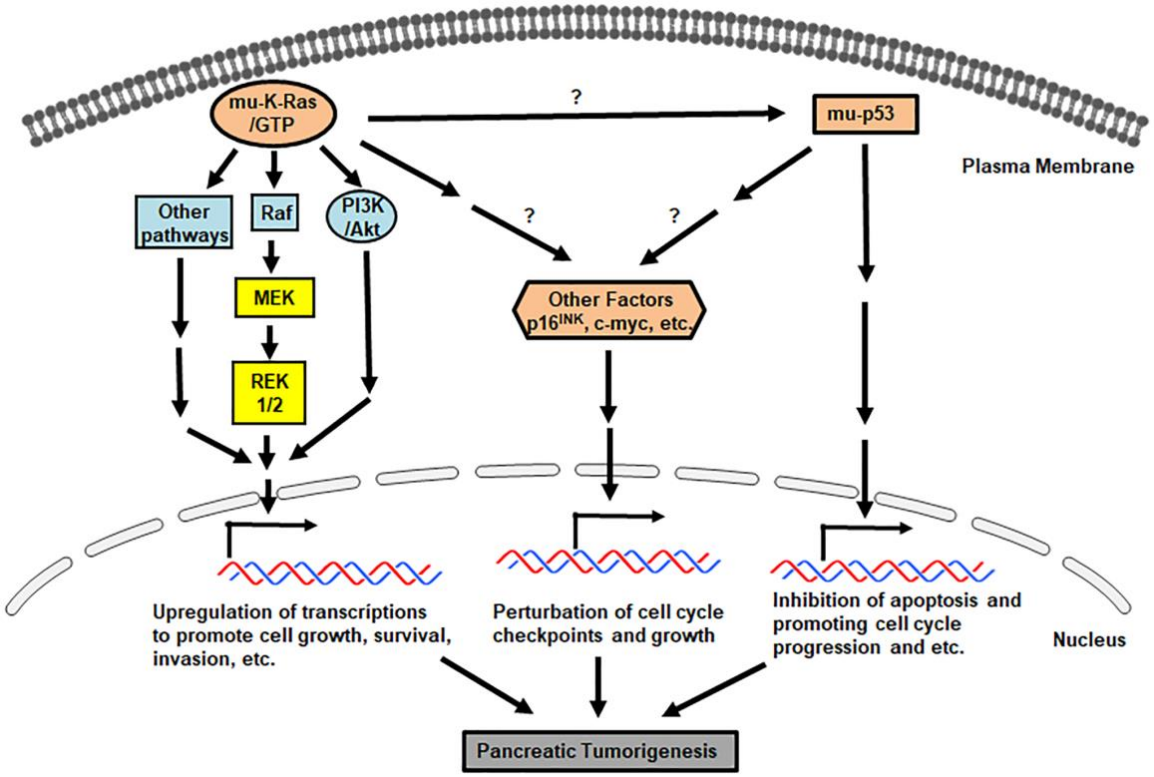
**Pro-pancreatic tumorigenic, aberrant genes/proteins and pathways.** Signals mediated by *mu-K-ras*, *mu-p53* and loss of *p16^INK^* promote various uncontrollable cell growth, cell cycle progression and etc.

Overall, it is clear that through precursor lesions, pancreatic tumors in some PanIN patients are evolved [16, 38, 45, 46]. Along with the progression of a clone carrying neoplastic precursors to malignancy, mutations of *K-ras* and tumor suppressors drive tumorigenesis, which worsens with arising genomic instability or genetic heterogeneity for malignant advancing [47–49]. A better understanding of molecular alterations in pancreatic cancer development should provide early diagnostic opportunities for recognizing primary cancer lesions as well as early onset periods for effective clinic interventions. Various growth-related signaling pathways driven by oncogenes and mutated genes are involved in promoting the formation of invasive pancreatic tumors. The major aberrant signaling pathways driven by these mutant factors in pancreatic cancer initiation and progression are summarized in Figure 1.

## Tumor microenvironment and immune suppression in pancreatic cancer

### Pancreatic cancer-associated fibroblasts

One of the features of pancreatic cancer is that the stroma is extremely desmoplastic infiltrative, which appears to account for its being highly resistant to currently available chemotherapies [50]. Stellate cells in the pancreas are myo-fibroblast-like cells or cancer-associated fibroblasts (CAFs) and the main component in tumor microenvironment that is composed of an extensive remodeling of the extracellular matrix (ECM) and cancer associated changes in the stroma [51, 52]. The tumor stroma is fibrotic and mainly contains fibrillar type I collagen and hyaluronan (HA). CAFs are a major player in building up fibrotic ECM. CAFs organize collagen fibrils to cause epithelial to mesenchymal transition (ETM) and further provide paths for cancer invasion. An increase of HA levels functions as an instructor directing cancer initiation and progression [53, 54].

Pancreatic stellate cells express vimentin, α-smooth muscle actin (αSMA) and desmin [51]. Via secretion of various growth factors, cytokines and chemokines, these CAFs act as safety guarders for cancer cell proliferation and invasion [55]. Pancreatic cancer stroma consisting of CAFs, ECM, vascular endothelial cells and immune suppressive regulatory T cells, functions as a barrier to protect cancer lesions and prevent drug penetration. It was demonstrated by that the co-injection of non-invasive pancreatic cancer cells with CAFs into rats, the cancer cells became metastatic [56]. Furthermore, the expressions of the matrix metalloproteinases (MMPs) in the surrounding of pancreatic cancer lesions are increased, which accelerate the degradation of collagen IV and laminin for ECM remodeling [57, 58]. Although roles of CAFs in pancreatic cancer development have not been fully understood, studies using cell lines and mouse models demonstrated that putative cells carrying CAF markers cooperated with cancerous cells, which played important roles in establishing anti-neoplastic drug resistance [54]. These CAF cells built up resistant barriers via secreting various cytokines, changing ECM structures, establishing cancer-associated metabolisms and inducing epigenetic and genetic instabilities [59]. Thus, it can certainly assume that cross talks between CAFs and pancreatic cancer cells are essential in the establishment of the tumor-prone microenvironment not only for facilitating the expansion of tumor masses, but also for preventing therapeutic interventions or establishing resistance to anti-pancreatic cancer treatments.

### Tumor suppressive lymphocytes

Another feature of pancreatic cancer is characterized by a highly immunosuppressive microenvironment that is built up by lacking intra-tumor effector T lymphocytes, existing mutant *K-ras*-driven oncogenic-prone inflammation, infiltrating suppressive immune cells and establishing the dense desmoplastic stromal reactions [16, 60, 61]. In order to build such cancer-prone environment, numbers of the effective immune cells [such as natural killer (NK) cells and CD8^+^ lymphocytes] are declined in pancreatic cancer lesions. On the contrary, regulatory T cells (Tregs), myeloid-derived suppressor (MDSCs) and tumor-associated macrophages (TAMS), together with CAFs, are significantly increased to seal the cancer cells from encountering the immune system.

T regulatory lymphocytes (Tregs) are suppressive T lymphocytes and can be recognized by their surface expressions of CD4^+^CD25^+^Foxp3^+^. Under normal conditions, Treg cells, via expressing CTLA-4 and secreting IL-10 or TGF-β, play important roles in inducing immune tolerance against auto-antigens, which prevents the occurrence of autoimmunity [59–61]. During tumorigenesis, Treg cells interfere with effector T cells to block immune responses against tumors. Pancreatic cancer cells, through chemokines or ligands/receptors, attract Tregs to translocate to their surrounding areas. For example, high levels of the ligands for chemokine receptor 5 (CCR5) were secreted by pancreatic cancer cells, which attract Tregs that express a fair amount of CCR5 [62, 63]. The interference of the association of the ligand and CCR5 prevented Treg migration to tumor lesions. Furthermore, TGF-β was shown to be required for recruiting Tregs to pancreatic tumor microenvironment [64, 65]. Higher populations of Tregs in pancreatic cancer lesions are strongly associated with poorer prognosis of the cancer patients.

### Cancer-associated macrophages

Macrophages are the phenotypically different innate immune cells derived from circulating monocytes in the blood stream and exist abundant in all tissues of the body. The terms of M1 and M2 of macrophages are described as their functional states. M1 macrophages are tumor destroyers that can be stimulated by Th1-associated factors or bacteria and express high amounts of IL-12. In contrast, M2 macrophages that are responsive to Th2-related cytokines and have high expression levels of IL-10 are immunosuppressive and in favor of tumor promotion. These two kinds of macrophages with the extremities of the polarization can be detected in a tumor lesion and exhibited high heterogeneities. Increases of M2 cells represent the progression of tumors, which often are accompanied with declining CD8^+^ and effector CD4^+^ T lymphocytes [66, 67]. By secreting matrix proteins and protease such as matrix metalloproteinases (MMPs), the tumor-associated macrophages promote metastasis of pancreatic cancer cells. In addition, by producing angiogenic factors, for example, vascular endothelial growth factor (VEGF) or Cox-2, and immunosuppressive lymphokines such as IL-10, the tumor-associated macrophages promote further development of an immunosuppressive environment [68, 69]. Tumor-associated macrophages express the programmed death-ligand 1 (PD-L1) on their surface, through which they interact with and induce effector T lymphocytes to undergo apoptosis for achieving their immunosuppressive function.

**Figure 2:**
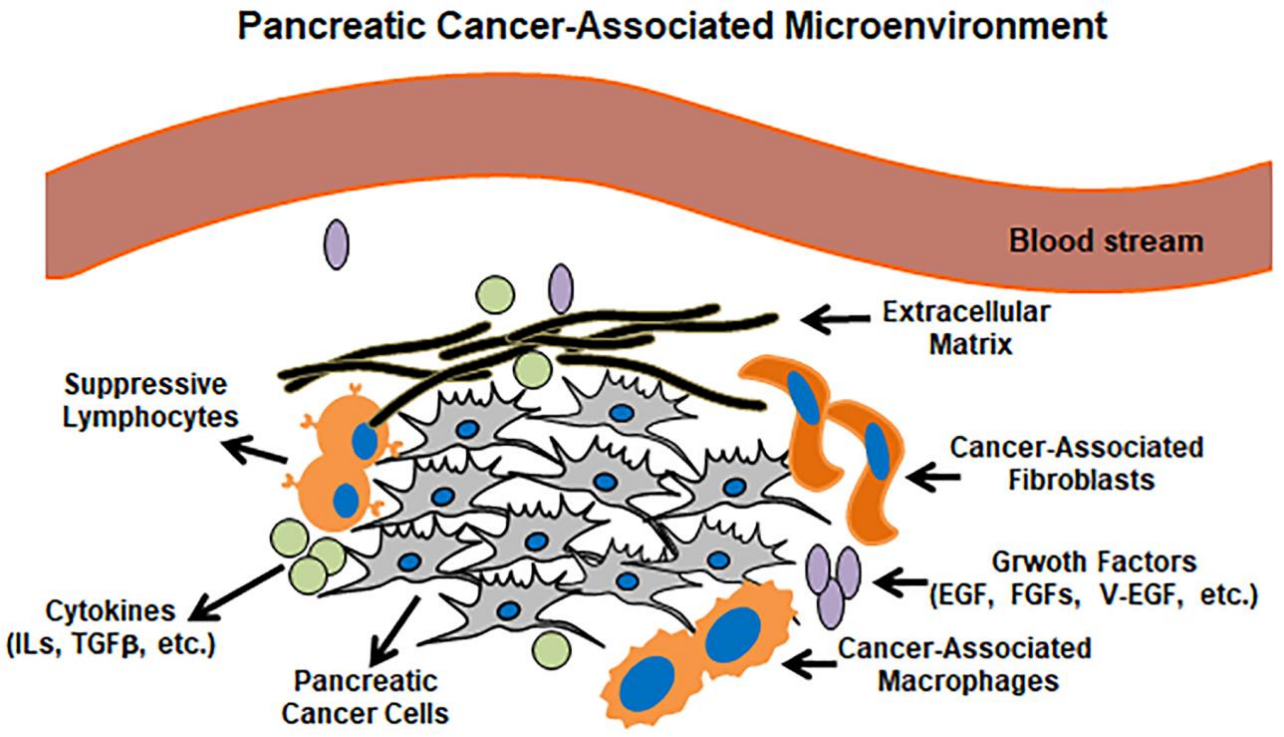
**Pancreatic cancer microenvironment.** Pancreatic cancer lesions mainly include pancreatic cancer cells, suppressive lymphocytes, cancer-associated macrophages/fibroblasts and cancer-related cytokines/growth factors.

In general, the profiles of tumor-associated microenvironment (TAM) are much more complex than we expect. TMA is infiltrated by dense stroma and functions pivotally in attracting cancer-related, various types of cells. Although tumor lesions exhibit different TAM phenotypes and are much more complicated, we here in a simplified way summarize a pancreatic tumor surrounded by a passive microenvironment barrier (Figure 2). Indeed, the tremendous plasticity of macrophages and TAMs provide a great therapeutic challenge, but also emphasize the urgent needs for developing TAM-targeted therapies.

**Figure 3:**
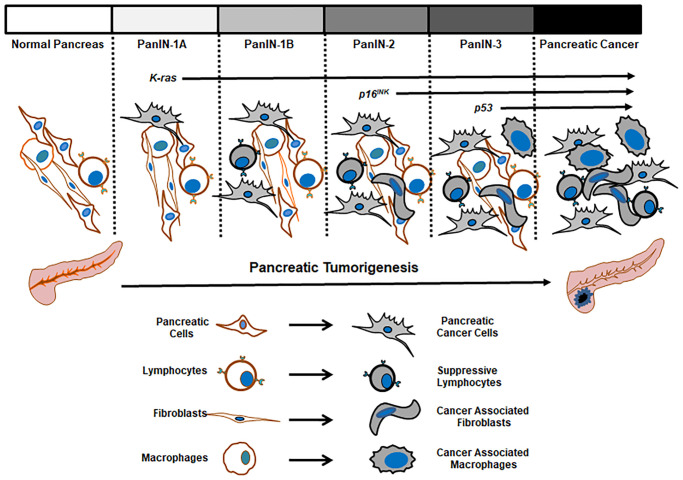
Pathological changes in the progression of pancreatic tumors.

Overall, from many of *in vivo*, *in vitro* and in patient studies, the classification of pathological lesions in conjunction with changes in genome, epigenome and microenvironment provides vast information to construct the accepted scheme of pancreatic tumor initiation and development from normal pancreases to PanIN, tumors *in situ* and further metastatic ones (Figure 3). Importantly, mutant *K-ras* activated by single point mutations can be detected during PanIN stages and appears a crucial element in promoting the onset of pancreatic cancer. Notably, some studies indicate that not all PanIN patients expressing mutant *K-ras* develop the cancer, which may be due to lack of occurrences of or coordination from other genetic or epigenetic changes [16, 70]. Therefore, more thorough investigations will shed lights into the role of *mu-K-ras* in pre-cancer stages and identify its cooperators in promoting pre-cancer cells to cancerous status.

## Current therapeutics for pancreatic cancer

### Surgery

Surgery is currently the only potentially curative treatment for pancreatic cancer patients, but only about twenty percent of the patients appear to reach a questionable “cure”. Most of pancreatic tumors often progress to either metastatic condition or to locally advanced situation for further radical resection [71, 72]. The availability to classify the tumors as resectable, borderline resectable, locally advanced pancreatic cancer (LAPC) or metastatic, significantly improves outcomes of the surgical treatment and helps make decisions for other alternative treatments [72].

The conventional surgery using in removing pancreatic tumors is the pancreatoduodenectomy by resecting the distal stomach, the pancreas head, the bile duct plus the duodenum. For patients with lesions in the pancreatic body or tail, distal pancreatectomy is employed, which is frequently in conjunction with splenectomy. When the tumor is extensively growing in the pancreas, total pancreatectomy can be necessary to achieve a radical resection. Overall, the 5-year survival rate following surgery is dismal with less than 20% and however, when one of the adjuvant chemo-treatments is administered, the survival rate can be somewhat improved [72].

### Pancreatic cancer chemotherapeutics

With a surgery aiming for cure, the recurrence rates of pancreatic cancer still are very high. Therefore, chemotherapy is the next, inevitable choice for patients after the operations. Nevertheless, the overall prognosis of patients undergoing adjuvant chemo-treatments remains dismal due to the low vasculature and building-up immunosuppressive cancer-associated microenvironment around the pancreas. Such barriers prohibit effective drug delivery or penetration, which appears less concern for patients receiving post-operative adjuvant treatments [72, 73].

Gemcitabine and its combination with other drugs are often used in pancreatic cancer chemotherapy. Gemcitabine is the first agent approved by the FDA for treating pancreatic cancer patients [74]. The known anti-cancer mechanisms of gemcitabine are primarily via generating the metabolite dFdCDP that inhibits ribonucleoside reductase and blocks DNA synthesis. This chemo-drug was initially used for treating virus infection and has then been implemented in anti-cancer therapy for solid tumors, including pancreatic cancer. Although its effect on overall survival rates is modest, its clinical beneficial response appears more positive than other drugs. However, the lack of the achievement of its survival benefits can be attributed to the establishment and development of cancer microenvironment in progressive cancerous lesions, which reduces or blocks persistent drug penetration [74].

5-Fluorouracil (5-FU) is another important anti-cancer drug. As an antimetabolite pyrimidine analogue, 5-FU promotes the incorporation of fluorouridine triphosphate into RNAs, of fluorodeoxyuridine triphosphate into DNAs as well as suppression of thymidylate synthase, resulting in severe genomic damages for eliminating cancer cells [75]. The treatment of this drug alone shows diverse ranges of the responses in pancreatic cancer patients [75, 76]. 5-FU is frequently used in combinations with other anticancer-drugs, including gemcitabine, cisplatin, doxorubicin or others [77]. Like gemcitabine, outcomes of the combination treatment did not show significant benefits for the patients as the single treatment. The clinical studies showed the relatively encouraging results, in which both the response rate of the co-treatment of 5-FU with cisplatin and the survival period are modestly improved in patients suffered from metastatic pancreatic cancer.

Folfirinox is a relatively effective, but aggressive combination treatment and a standard therapy for pancreatic cancer patients. This combined treatment is composed of 4 existing anticancer-drugs: 5-FU, irinotecan, oxaliplatin and leucovorin and offers an objective response rate of 32% with a median progression-free survival of 6 months for pancreatic cancer patients [78, 79]. However, this treatment has some side effects presented as fatigue, bone marrow suppression with neutropenia, gastrointestinal disorder and sensory neuropathy. Currently, modified Folfirinox has been implemented in clinic only for those patients who are after surgery and relatively fitted or tolerable to the treatment.

### Immunotherapy

As mentioned above, a solid fibrotic and cancerous microenvironment is rapidly formed around pancreatic cancer lesions, which prevents drug penetration and at the same time, suppresses immune reactions. Immunosuppressive cytokines (such as IL-6 or others) that are produced by surrounding tumor stroma are augmented in the tumor lesions [74, 80]. These secreted cytokines, including arginase-I, reactive oxygen species (ROS) and suppressive cytokines (IL-10, TGF-β and others), stimulate further expansion of immunosuppressive lymphocytes and other cancer associated populations to antagonize anticancer responses. Under such suppressive influences, the body antitumor immune-responses are further mitigated and at the same time, inhibitory dendritic cells (DC), Tregs and cancer-associated macrophages are accumulated. With these vicious feedback and feedforward stimulations, a solid tumor associated microenvironment is built to keep chemo-drugs from reaching pancreatic cancer lesions [74]. Notably, immunotherapy is encouraging, but more complex than we anticipate. Thus, it is imminent for developing strategies to target immune checkpoints that are perturbed by cancerous cells. Programmed death-1 (PD-1) and cytotoxic T lymphocyte antigen-4 (CTLA-4) are well known immune checkpoints among others [80, 81]. Ipilimumab is an anti-CTLA-4 antibody and the first FDA-approved, antibody-based treatment [82]. The drug is shown to improve the overall survival of pancreatic cancer patients. Nivolumab and pembrolizumab (the PD-1 inhibitors) are used clinically for treating melanoma and still under clinical investigations for treating pancreatic cancer [83]. In the animal study, it was also shown that the combined inhibition of IL-6 and PD-1 enhanced numbers of tumor infiltrating T lymphocytes [84]. Therapeutics against potentially mitigating immunosuppressive cells to reach pancreatic tumor microenvironment appeared to enhance efficacy of immune-based therapies. Because pancreatic tumors possess the immune-advantaged nature at early stages of the initiation, which equip them to escape immune surveillance, currently existing immunotherapies have not demonstrated breaking-through results in this aspect yet.

### Other approaches for pancreatic cancer therapy

#### Inhibition of hyaluronan for pancreatic cancer treatment

Pancreatic tumor associated microenvironment is composed of various types of cells that play crucial roles in remodeling surrounding normal tissues and promoting EMT, through upregulating metalloproteinases and cytokines to change the extracellular matrix to fibrotic surrounding to antagonize chemo-drugs and to promote pancreatic tumorigenesis [50]. As a glycosaminoglycan, hyaluronan is a major element in cancer extracellular matrix, increases of which are associated with poor prognosis of pancreatic cancer patients. Therefore, PEGPH20 (a pegylated hyaluronidase) among other derivatives that can break down hyaluronan in tumor microenvironment barriers has been investigated clinically. The combination of gemcitabine, nab-paclitaxel and PEGPH20 is in the phase II of clinical trials and however, the overall survival rates of unselected patients did not show a strong improvement [85]. Notably, hyaluronan derived drugs are more toxic. Therefore, studies for developing hyaluronan related drugs have been only focusing on the pancreatic cancer patients expressing high amounts of hyaluronan.

#### Other targeting therapeutics

Mutant *K-ras* is a potential drug target and however, inhibitors directly against this onco-protein have not been successful, due to the complexity of Ras involved in regulating multiple downstream effectors and interconnecting with various parallel signaling pathways. Recently, a set of small molecules that can covalently bind to the switch-I and –II pockets of mutant K-ras^G12C^ provides the hope in treating cancers harboring oncogenic *K-ras* Several candidates, such as AMG510, ARS3248 and MRTX849, are generated and currently in clinical trials for treating solid tumors like pancreatic, colon and lung cancers [86]. Among these candidates, AMG510 (sotorasib, LUMAKRAS™) is the first one approved by FDA for treating *K-ras^G12C^* lung cancer patients.

Co-expression of mutant *K-ras* and *p53* exists in most of pancreatic cancer. Therapeutic strategies of introducing *wt-p53* into cancer cells have been developed. The research showed that the re-introduction of *wt-p53* was able to enhance cytotoxicity of gemcitabine or temozolomide for eliminating pancreatic tumors [87]. Such approaches also generated a profound growth suppression of pancreatic cancer cells. Thus, gene therapies to restore of *wt-p53* in cancers, including pancreatic cancer, are promising and currently in clinical trials.

Aberrant activation of hedgehog pathway functions to support the viability of cancer stem cells and formation of tumor stroma, and has been the target for developing effective therapeutics. Cyclopamine is an inhibitor of the formation of cancer stroma and the role of this drug in treating pancreatic cancer has been explored [88]. The combination of vismodegib (a cyclopamine derivative) with gemcitabine was shown to slightly increase the overall survival rates in pancreatic cancer patients [88]. Saridegib (another drug of cyclopamine derivative) in combination with folfirinox is in the phase I of the clinical trials and shown a better result [89].

The signaling pathway mediated by the Janus kinase and activator of transcription (JAK/STAT) is suggested to be involved in inducing inflammation in host tissues and tumor lesions. STAT3 is one of STAT proteins and a key element in promoting the growth of pancreatic tumors harboring oncogenic *K-ras*. In the early clinical trial studies, napabucasin (a JAK/STAT inhibitor-based drug) was shown to be very encouraging in treating pancreatic cancer [90]. In addition, the combination treatment of AZD-1950 (an antisense-STAT3 based drug) and durvalumab currently enters the phase II of the clinical trials for pancreatic cancer patients (NCT02983578). Importantly, in these primary clinical studies targeting JAK/STAT signaling pathway, no obvious toxicity is observed. Thus, the clinical trials to develop drugs inhibiting JAK/STAT pathway are actively moving forward.

## Summary

Currently, chemo-drugs (for example, gemcitabine) and their combinations with other anticancer drugs or treatments are still the standard regimens for pancreatic cancer. Local administration of chemo-drugs appears to provide bearable and better therapeutic effects than the systemic approaches. The development of new molecular technologies for the identifications of targeted genes and proteins has been providing the promises in helping discover novel therapeutics for this devastating malignancy. In particular, tumor genetic profiles, in conjunction with other newly developed technologies have permitted better clarifications of pancreatic cancer lesions and prognostic markers as well as more initiatives for producing effective drugs. All these will set the stage for improving the prognosis for pancreatic cancer patients.
